# Prevalence of* Toxoplasma gondii*,* Neospora caninum*, and* Sarcocystis* Species DNA in the Heart and Breast Muscles of Rock Pigeons* (Columbia livia)*

**DOI:** 10.1155/2018/6264042

**Published:** 2018-05-06

**Authors:** Muhammad Mudasser Nazir, Muhammad Mazhar Ayaz, Atif Nisar Ahmed, Azhar Maqbool, Kamran Ashraf, Muhammad Oneeb, Ghulam Yasin, Atta Subhani, Muhammad Asif Ali, Noman Nazir, Muhammad Afzal Sajid

**Affiliations:** ^1^Department of Pathobiology, Bahauddin Zakariya University, Multan 60800, Pakistan; ^2^Department of Parasitology, University of Veterinary and Animal Sciences, Lahore 54600, Pakistan; ^3^Institute of Molecular Biology and Biotechnology, University of Lahore, Lahore 54500, Pakistan; ^4^Camel Breeding and Research Station, Rakh Mahni, Bhakkar 30000, Pakistan; ^5^Department of Food and Nutrition, University of Veterinary and Animal Sciences, Lahore 54600, Pakistan; ^6^Shifa International Hospital, Islamabad 44000, Pakistan; ^7^Veterinary Research Institute, Lahore 54810, Pakistan

## Abstract

Little is known about the prevalence of protozoan parasites in the muscles of rock pigeons* (Columbia livia)*. The muscles from 54 (heart from 45 and breast from 54) rock pigeons were examined for DNA of* Toxoplasma gondii*,* Neospora caninum, *and* Sarcocystis *species using PCR. Twenty-four were female and 30 were males. The birds were part of flocks of pigeons housed at the tombs of saints in Lahore, Pakistan. Birds that died or were euthanized due to poor health were submitted for necropsy at the Department of Parasitology, University of Veterinary and Animal Sciences, Lahore, Pakistan, where DNA isolations and PCR were conducted. Nineteen (35.1%) of the birds were positive for* T. gondii* DNA. Seven males and 12 females were positive. Breast tissue was always infected in* T. gondii* positive birds, while the heart was infected in 13 (28.8%) of breast positive birds. Five (9.2%) of the pigeons, 2 males and 3 females, were positive for* N. caninum*. The distribution of* N. caninum* DNA was more variable in the muscles of pigeons than* T. gondii *and was found only in the heart of 1 (female), heart and breast muscle of 2 (male), and only the breast muscle of 2 birds (female). One of the 54 rock pigeons (female) was positive for both* T. gondii* (heart and breast) and* N. caninum* (heart only). Two of the positive* Neospora caninum* amplicons were sequenced and had 97% nucleotide identity with* N. caninum *isolates.* Sarcocystis* DNA was not found in any bird. The prevalence of* T. gondii* in rock pigeons and their predation by cats suggest that they may play an unrecognized role in maintaining environmental contamination with* T. gondii* oocysts by cats. Our study indicates that rock pigeons are intermediate hosts of* N. caninum* and this information will aid in understanding the epidemiology of* N. caninum*.

## 1. Introduction

*Toxoplasma gondii*,* Neospora caninum*, and* Sarcocystis* species are apicomplexan parasites that are important parasites of humans and domestic and wild animals. These protozoans use intermediate hosts in their life cycles and produce tissue cysts in the intermediate host that are infectious for an appropriate definitive host. Tissue cysts of these parasites can be identified using morphological, biological, and molecular methods. We are interested in examining the epidemiology of encysted apicomplexan parasites to gain insight into measures to limit transmission of these parasites. Rock pigeons,* Columbia livia,* are present throughout the world and there are no reported surveys on the prevalence of encysted apicomplexan parasites in the muscles of rock pigeons. Rock pigeons are not primarily carnivorous, so any infections in these hosts would be due to oocyst or sporocyst acquired infections. The present study reports the results of our study to detect the tissue cysts of* T. gondii*,* N. caninum*, and* Sarcocystis* species in the heart and breast muscles of rock pigeons.

## 2. Materials and Methods

### 2.1. Birds and Tissue Samples

The birds were part of flock of pigeons housed at the tombs of saints in Lahore, Pakistan. Birds that died or were euthanized due to poor health were submitted for necropsy at the Department of Parasitology, University of Veterinary and Animal Sciences, Lahore, Pakistan, where DNA isolations and PCR were conducted. The muscles from 54 (heart from 45 and breast from 54) rock pigeons were examined for DNA of* T. gondii, N. caninum, S. neurona/S. falcatula*, and* Sarcocystis* species using PCR. Twenty-four were female and 30 were males.

### 2.2. Muscle Sample Collection and PCR

Approximately 0.5 g sample of breast muscle from each of the 54 birds and the heart from 45 birds was removed and frozen at −20°C until being used for DNA isolation. Necropsy instruments were washed between each tissue and between birds. DNA was extracted using a commercial DNA extraction kit (Isolate Genomic DNA Kit, Bioline, Taunton, Massachusetts, USA) according to the manufacturer's instructions.

PCR was conducted for* T. gondii* using the primers (TOX4/TOX5) described by Homan et al. [1] for the multicopy B1 gene. DNA collected from RH strain tachyzoites were used as positive controls for* T. gondii *PCR. PCR for* N. caninum* was conducted using the primers Np6/Np21 described by Yamage et al. [2]. DNA collected from NC-1 strain tachyzoites were used as positive controls for* N. caninum *PCR. PCR for* Sarcocystis* species was done using the ITS-1 primers 18S9L/18S1H described by Li et al. [3] and PCR for* S. falcatula/S. neurona* was done using the primers JNB33/JNB54 described by Tanhauser et al. [4]. DNA collected from merozoites of the SF-1 strain of* S. falcatula* or SN6 strain of* S. neurona* were used as positive controls for* Sarcocystis* PCR [5].

PCR were performed in 25 *μ*l volumes containing the primer pairs and the template in a commercial PCR Master Mix (Promega, Madison, Wisconsin, USA). PCR cycling conditions described for each primer pair and parasite were used. Nuclease-free water was used as a negative control, parasite specific DNA was used as a positive control (see above), and 100 bp DNA ladder (Invitrogen, Carlsbad, CA, USA) was run to determine the kb of the PCR products.

### 2.3. Sequencing* N. caninum* Amplicons

DNA bands corresponding to positive samples for* N. caninum* were excised with a fine sterile punch and the amplicons were extracted using the QIAquick Gel Extraction Kit (QIAGEN Sciences). The extracted DNA were submitted for sequencing to the Institute of Biochemistry and Biotechnology, UVAS, Lahore, where PCR products were directly sequenced in the forward and reverse directions using the Big Dye terminator system, version 3.1 (Applied Biosystems), and an ABI 3130xl sequencer (Applied Biosystems). The sequence chromatograms were edited using BioEdit Sequence Alignment Editor, version 7.0.9.0 (Ibis Biosciences, California, USA). BLAST searches were performed in order to compare the sequences with those in the public database.

### 2.4. Ethics

All the bids investigations were executed in accordance with procedures ratified by each institution.

## 3. Results

### 3.1. Prevalence

Nineteen (35.1%) of the birds were positive for* T. gondii* DNA: 7 were males and 12 were females (Table 1). Breast tissue was always infected in* T. gondii* positive birds, while the heart was infected in 13 (28.8%) of the 45 rock pigeons which had both tissues examined. Five (9.2%) of the rock pigeons were positive for* N. caninum *(Table 1). The distribution of* N. caninum* DNA was heart of 1 (female), heart and breast muscle of 2 (male), and only the breast muscles of 2 birds (female). One of the 54 rock pigeons (female) was positive for both* T. gondii* (heart and breast) and* N. caninum* (heart only). No birds were positive for* Sarcocystis* species DNA in their muscles.

### 3.2. *N. caninum* Sequencing

Two of the positive* N. caninum* sequenced amplicons indicated that there was 97% homology of the sequences obtained from birds in the present study with the NC-1 isolate of* N. caninum*.

## 4. Discussion

Clinical and subclinical toxoplasmosis have been observed in pigeons and other avian species like Passeriformes including canaries* (Serinus canarius)*, greenfinches* (Carduelis chloris)*, goldfinches* (Carduelis carduelis)*, sirkins* (Carduelis spinus)*, Linnets* (Carduelis cannabina)*, bullfinches* (Pyrrhula pyrrhula)*, Hawaiian crow* (Corvus hawaiiensis)*, satin bowerbirds* (Ptilonorhynchus violaceus)*, regent bowerbirds* (Sericulus chrysocephalus)*, and red-whiskered bulbuls* (Pycnonotus jocosus)* [6]. Our study demonstrates that rock pigeons are often infected with* T. gondii*. They probably consume paratenic hosts in the form of insects that have acquired infection or have directly picked oocysts up from feeding on the ground. They do not spend a majority of their time feeding from the ground as do chickens which are considered good indicators of soil contamination with* T. gondii* [6].

Molina-López et al. [7] reported that 24 of 67 (36%) common ravens* (Corvus corax)* from Spain were positive for antibodies to* N. caninum* in an IFA test. Baker et al. [8] were not able to demonstrate* N. caninum* infections in carnivorous birds including red-tailed hawks* (Buteo jamaicensis)*, turkey vultures* (Cathartes aura)*, barn owls* (Tyto alba)*, or American crows* (Corvus brachyrhynchus)* fed rodents chronically infected with* N. caninum*. However, the goal of that study [8] was to determine if birds were the definitive host of* N. caninum* and their tissues were not examined for* N. caninum*. Our finding of* N. caninum* DNA in rock pigeons is consistent with the finding of* N. caninum* DNA in other birds and Passeriformes (sparrows, magpies, and ravens) suggesting this order of birds may be useful in examining the epidemiology of* N. caninum*. Domestic and wild canids will consume birds and the potential of these birds to be sources of* N. caninum* for canids and the resulting environmental contamination with oocysts excreted by these canids are an area of research that has not yet been explored. The results of DNA sequencing indicate that the identity of* N. caninum* is consistent with the NC-1 isolate of the parasite isolated from dogs.

We looked for DNA of* S. falcatula* (and* S. falcatula*-like) because this parasite has been found in many species of birds from different orders [9–11]. They have been observed frequently in outbreaks of disease in captive birds [12, 13] and are emerging as a pathogen of raptors [14–18] and other wild birds [19–21] in North America. By using primers JNB33/JNB54, we were looking for DNA of* S. falcatula*,* S. neurona*, or* S. falcatula*-like parasites that might be present in birds and by using ITS-1 primers 18S9L/18S1H we were looking for* Sarcocystis* species other than the opossum transmitted* S. falcatula*-like parasites. None of the rock pigeons examined had indication of infection with any* Sarcocystis* species. However the lack of* Sarcocystis* species infection in rock pigeons may be due to absence of definitive host in this area.

## Figures and Tables

**Table 1:** Prevalence of apicomplexan parasites in heart and breast muscles of rock pigeons.

Parasite	Samples	Sex	Number of samples (*n*)	Positive samples (*n*)	Sex-wise prevalence	Organ-wise prevalence	Overall prevalence
*T. gondii*	Breast positivity rate	Male	30	7	23.3%	35.1%	35.1%
		Female	24	12	50%		
	Heart positivity rate	Male	23	5	21.7%	28.8%	
		Female	22	8	36.4%		
	Heart & breast positivity rate	Male	23	5	21.7%	28.8%	
		Female	22	8	36.4%		

*N. caninum*	Breast positivity rate	Male	30	2	6.6%	7.4%	9.2%
		Female	24	2	8.3%		
	Heart positivity rate	Male	23	2	8.6%	6.6%	
		Female	22	1	4.5%		
	Heart & breast positivity rate	Male	23	2	8.6	11.1%	
		Female	22	3	13.6		
